# Medical News and Miscellany

**Published:** 1899-12

**Authors:** 


### Medical News and Miscellany.

**Change of Address.**—Dr. E. A. McMahan from Marshall, Tex., to Tolar, Tex.; Dr. B. E. Hadra from San Antonio to Dallas, Tex.

**Married,** in San Antonio, Tex., Nov. 29 (ult.), Dr. John W. Kenney, formerly of Cook's, N. M., but now of San Antonio, to Miss Lillie Vance of that city. No cards.

**The New Orleans Polyclinic,** thirteenth annual session, opens November 20, 1899; closes May 10, 1900. Every inducement in clinical facilities for those attending. The specialties are fully taught. Further information, New Orleans Polyclinic, New Orleans, La.

**A $3000 Dollar Practice** given away to the purchaser of my property, consisting of a residence, outhouses, lots, office and stock of drugs; all located in a village in the black lands of central Texas. No competition. Will introduce successor. Price of all $1000, half cash, balance in twelve months. Address Dr. C. T., care Texas Medical Journal.

**Infantile Constipation.**—Dr. L. Emmett Holt has said that the conditions found in young children were such as to make the treatment of constipation in these little patients proportionately easy and satisfactory. He always insists upon a careful scrutiny of the whole of the child's daily life. The nurse was often to blame for the constipation in the infant. In infants under six months of age constipation was often due to modified milk in too small percentages, so that too little residue was left. Under such circumstances the indication was to increase the total solids. The bad cases of chronic constipation in children of five or six years of age that he had seen had been almost without exception in children who were exceedingly healthy and robust. He could heartily recommend cascara as being probably the most valuable drug in the treatment of cases in which there was loss of expulsive power. Still better than drugs, in his opinion, were suppositories.—*Medical Record.*

### "Functional" Heart Murmurs.

Dr. A. Jacobi (*American Medical Quarterly*, September), thus concludes a paper on this subject:

"1. The diagnosis of deranged function in any organ is only a makeshift, and is justifiable only so long as we are ignorant of the physical cause of that derangement. That heart murmur is called 'functional' the anatomical cause of which we do not know. That is why a skilled diagnostician may recognize fewer functional murmurs than one who will not diagnosticate a heart disease unless he has all the symptoms, including dilatation and hypertrophy.

"2. The same disorders of the blood and nervous system in which heart murmurs are observed in the adult, do not cause them in the small infant. In the latter the heart is larger, more robust, and more powerful, and its contractions are more uniform and effective; its two ventricles are equally muscular, or nearly so, and the valves are smaller. Thus, the greater frequency of murmurs in the adult is attributable to the physical condition of his heart, and should not be explained by a deranged function.

"3. Even in the present limitation of our knowledge we should agree to call 'functional' only those murmurs which are temporary, or intermittent, or variable in their character. They are met with in the neurotic and neurasthenic, in the (adult) anæmic, sometimes in syncope or in chorea minor, and occasionally in rheumatism. Even here they should be recognized as myocardial or as neurotic."
—*New York Medical Journal.*

### The Immorality of Christian Science.

"Fools rush in where angels fear to tread."

Knowing nothing of disease, and virtually denying its existence, it should scarcely occasion surprise that the self-styled "Christian Scientist" should boastfully proclaim his ability to cure the sick and heal the maimed. So deluded have some of the disciples of the cult become, that they are permitting their zeal to carry them beyond the bounds of possibility. With their beliefs we have nothing to do, but when they promulgate the hope, as did a public speaker recently in Philadelphia, that "cancer, consumption, Bright's disease, blindness, typhoid fever, curvature of the spine, locomotor ataxia, valvular disease of the heart, and the alcohol and drug habits" can be cured by "Christian Science," tolerance is exhausted, and steps should be taken to restrain the rabid utterances and the irrational practices of such ignorant and irresponsible persons. Liberty is one thing, and license another, and the crime of even suggesting such obviously false doctrines and immoral practices should be prevented by severe punishment. Those who speak thus should know better, or their ignorance is criminal. Human life must still be considered as too serious and too valuable to be thus lightly dealt with.—*Journal American Medical Association.*

---

### Selected Formulae.

---

**Cardiac Stimulation for a Child.**—

| | | |
|---|---|---|
| ℞ | Spir. etheris comp................. | ♏ 80 |
| | Tinct. nucis vomicæ........ ..... | ♏ 40 |
| | Tinct. lavandulæ comp ... ..... | ♏ 80 |
| | Aq. cari ............................q. s. ad | 3 4 |

M. Sig. One tablespoonful every four hours to a child of eight to twelve years old.—*Ashby*, *Medical Record.*

---

**Iodine in Treatment of Chronic Eczema of the Hands.**—The *Revista de Medicina y Cirugia Practicas*, quoting the *Therapeutische Monatshefte*, attributes the following formula to Edlefsen:

| | | |
|---|---|---|
| ℞ | Iodine.......................................gr. | 1½ |
| | Potassium iodide.......................gr. | 4 |
| | Glycerine....................................gr. | 180 |

M. Sig. To be applied every night and the hands covered with compresses.—*N. Y. Medical Journal.*

---

**Itch Ointment.**—In a series of experiments at the St. Luke's Hospital, Paris, to determine what will cure itch in the shortest time, forty-one different preparations were employed. One of these the following ointment cured in the smallest number of days:

| | | |
|---|---|---|
| ℞ | Sublimated sulphur....................... | ℥ ij |
| | Subcarbonate of potash................. | ℥ j |
| | Adeps simplex............................ | ℥ viij |

M. Sig. Apply morning and night.

The writer of this has been in the habit of adding to the above the oil of bergamont, three drams, thus adding to the fiavor and potency of the ointment.—*Modern Medicine.*

